# For Regulating Teeth

**Published:** 1894-04

**Authors:** J. F. Steele

**Affiliations:** Estherville, Iowa


### For Regulating Teeth.

BY J. F STEELE, D.D.S., ESTHERVILLE, IOWA.

A very cheap appliance can be made for regulating teeth, and one that will do the work in all cases with tools and materials that every dentist has in his office, at least he should have. The articles are as follows: Silver composition strips, invention of Dr. Parmly Brown, made by S. S. White Co., one dozen in box, price fifty cents per box; the next is the Stub's Screw Plate and Taps, made in England, can be got of any dental depot, one dollar and fifty cents.

Select a strip the proper width, wrap it around the teeth you want to anchor to, make a screw and nut of No. 20 American standard gauge wire, flatten the wire and cut off a piece for the nut, make a hole in one end of bolt, make a fine wire loop, put through hole in bolt, pass it over the tooth that you want to draw in place; now, drill a hole in the metal strip opposite the position you want the tooth to take; pass the bolt through and put on your bur and pull as long and strong as you please. I have drawn teeth five-eights of an inch in three weeks with this system. I have used it successfully in over twenty-five cases; try it, you will be highly pleased with it, I am sure.

I am preparing an article on making crowns of plate teeth, that is, pivot crowns; also one on bridge-work, combination of metal and rubber. I have some cases of bridge-work that have stood the test for ten years.

